# A novel missense mutation in *P4HB* causes mild osteogenesis imperfecta

**DOI:** 10.1042/BSR20182118

**Published:** 2019-04-30

**Authors:** Lujiao Li, Dichen Zhao, Wenbin Zheng, Ou Wang, Yan Jiang, Weibo Xia, Xiaoping Xing, Mei Li

**Affiliations:** Department of Endocrinology, National Health Commission Key Laboratory of Endocrinology, Peking Union Medical College Hospital, Chinese Academy of Medical Sciences and Peking Union Medical College, Beijing 100730, China

**Keywords:** bisphosphonates, novel mutation, osteogenesis imperfecta, P4HB

## Abstract

Osteogenesis imperfecta (OI) is a rare heritable bone disorder characterized by low bone mineral density (BMD), recurrent bone fractures, and progressive bone deformities. *P4HB* encodes protein disulfide isomerase (PDI) and is identified as a novel candidate gene of OI. The purposes of the present study are to detect pathogenic mutation, to evaluate the phenotypes of a Chinese family with mild OI, and to investigate the effects of bisphosphonates on bone of the proband. We detected the pathogenic mutation by next generation sequencing and Sanger sequencing. Laboratory and radiological investigations were conducted to evaluate the phenotypes. The proband was a 12-year-old girl with low BMD, history of recurrent non-traumatic fractures, slight scoliosis, with bluish grey sclera and ligamentous laxity. Her father suffered from one fragility fracture and slight wedge changes of vertebras, with bluish grey sclera. We identified a novel heterozygous missense mutation (c.692A>C, p.His231Pro) in *P4HB* in the proband and her father. This mutation was predicted to affect the combination of PDI with type I procollagen and lead to the disorder of its triple helix formation. Bisphosphonates were effective in reducing bone resorption and increasing BMD of the proband with well tolerance. In conclusion, we identified a novel mutation in *P4HB* in a Chinese family with mild OI, which expanded the genotypic and phenotypic spectrum of OI. Bisphosphonates were effective to this extremely rare OI induced by *P4HB* mutation.

## Introduction

Osteogenesis imperfecta (OI) is a rare heritable bone disorder with an incidence of 1:15,000–20,000 neonates, which is characterized by low bone mineral density (BMD), impaired bone strength, resulting in recurrent bone fractures and progressive bone deformities [1]. Patients with OI also present with a series of extra-skeletal manifestations, including dentinogenesis imperfecta, blue sclera, hearing deficits, and ligamentous laxity [2]. The phenotypes of OI widely vary from mild to perinatal death, so there are four main clinical categories of OI (types I–IV) [3]. With the development of molecular diagnosis, additional types of OI have been found. Up to now, at least 20 casuative genes of OI have been identified, including *COL1A1, COL1A2, IFITM5, CRTAP, LEPRE1, FKBP10, PLOD2, PPIB, SERPINF1, SERPINH1, SP7, WNT1, BMP1, TMEM38B, PLS3, CREB3L1, SEC24D, SPARC, P4HB*, and *MBTPS2*, which are involved in encoding or post-translational modification process of type I collagen or regulating osteoblasts function [4–9].

Recently, *P4HB* (OMIM 176790) is reported as a new candidate gene of a severe type of OI, Cole-Carpenter syndrome (CCS) [10–13]. CCS was first identified in 1987 by Cole and Carpenter, which was characterized by bone fragility, craniosynostosis, hydrocephalus, wide open fontanelle, ocular proptosis, blue sclera, small nose, flat nasal bridge, and other distinctive facial features [14]. *P4HB* locates on chromosome 17q25.3 and spans 18 Kb of 11 exons, which encodes protein disulfide isomerase (PDI), a key enzyme for protein folding by forming the correct disulfide bridges between polypeptide chains [15–17]. PDI plays an important role in post-translational modification of type I procollagen as a chaperone to prevent aggregation of procollagen α chains. And it is also the β-subunit of prolyl 4-hydroxylase that is responsible for hydroxylating proline residues on α chains of type I procollagen [18,19]. As far as we know, only two types of mutations in *P4HB* are reported, both of them caused CCS [10–13].

In the present study, we aim to detect pathogenic mutation and to investigate the phenotypes of a Chinese family with mild OI. In addition, we also prospectively evaluate the effects of bisphosphonates on bone of the proband.

## Methods

### Subjects

**Figure 1:**
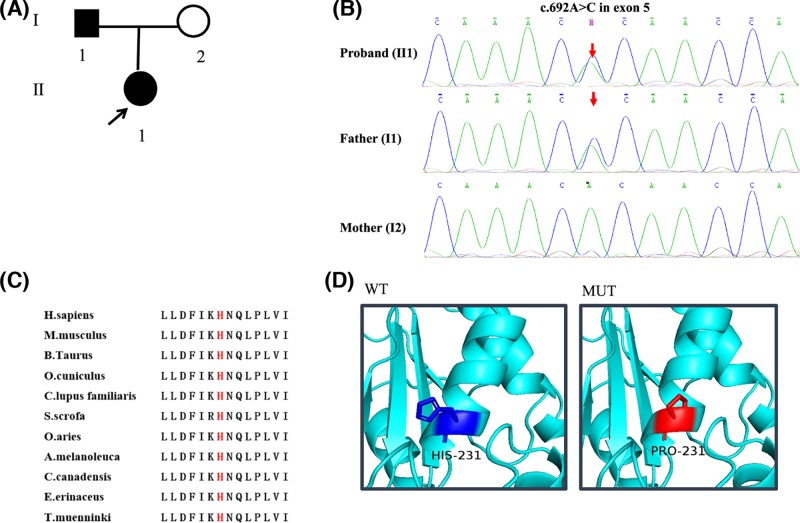
Verification and analysis of the mutation in *P4HB* (**A**) Pedigrees of the family in the present study. The proband was indicated by black arrows. (**B**) Sanger sequencing results of the proband and her parents. In the proband, novel mutation in *P4HB* was identified as c.692A>C in exon 5 (indicated by red arrows). (**C**) p.H231 residue in PDI (NP_000909.2) was highly conserved amongst 11 different species. (**D**) Close-up of the 3D structural model of the mutated position in PDI (WT). Position 231 was a histidine in normal population. (MUT) His231Pro led to a change in the last α helixes of the domain b of PDI indicated in red. MUT, mutant type; WT, wild type.

A 12-year-old Chinese girl of Han origin from a non-consanguineous family went to endocrinology clinic, Peking Union Medical College Hospital (PUMCH) in 2014 for 6 years history of recurrent fractures. She was diagnosed as OI in our clinic. The proband and her parents were included in the present study. The pedigree of this family was shown in Figure 1A.

The study protocol was approved by the Scientific Ethics Committee of PUMCH. The patient’s parents signed the informed consents before they participated in the present study.

### Evaluation of phenotype

Medical history was collected in detail, including fracture history, growth and development status, and family history. Meanwhile, physical examination was carefully performed, including bone, teeth, sclerae, and joints. Height and weight of the patient were measured with Harpenden stadiometer (Seritex Inc., East Rutherford, NJ, U.S.A.) and RGZ-120 weighing scale (Xiheng, Wuxi, China), which were calculated to age- and sex-specific Z scores on the basis of the reference data of Chinese children [20].

Serum levels of calcium (Ca), phosphate (P), alkaline phosphatase (ALP, a bone formation marker), aminotransferase (ALT), creatinine (Cr) were measured using an automated analyzer (ADVIA1800, Siemens, Germany). Serum levels of 25-hydroxyvitamin D (25OHD), β cross-linked carboxy-terminal telopeptide of type I collagen (β-CTX, a bone resorption marker) and intact parathyroid hormone (PTH) was quantitated by an automated electrochemiluminescence system (E170; Roche Diagnostics, Switzerland). Vitamin D deficiency, insufficiency, and sufficiency were defined as serum 25OHD level <20, 20–30, and >30 ng/ml, respectively [21]. All biochemical parameters were measured in the central laboratory of PUMCH.

Dual energy X-ray absorptiometry (DXA, Lunar Prodigy Advance, GE Healthcare, U.S.A.) was used to measure BMD at lumbar spine and femoral neck. BMD Z scores were calculated according to the normal reference data from age and gender matched population [22,23]. X-ray films of the skull, radius, ulna, hands, and thoracolumbar spine were examined.

### Detection of pathogenic mutation

Genomic DNA was extracted from peripheral leukocytes under the standard procedures (QIAamp DNA Mini Kit, Qiagen, Germany). Genetic detection was performed through paired-end sequencing using a panel for next generation sequencing (NGS), which covered more than 700 candidate genes of genetic bone disorders, including 20 candidate genes of OI (*COL1A1, COL1A2, IFITM5, SERPINF1, CRTAP, P3H1, PPIB, SERPINH1, FKBP10, BMP1, PLOD2, SP7, TMEM38B, WNT1, CREB3H1, SPARC, PLS3, P4HB, SEC24D*, and *MBTPS2*). Protocol of the experimental procedures was as previously described [24]. The genomic DNA was fragmented and ligated with end-repaired adaptors oligonucleotides. Then DNA fragments with adaptor molecules were purified and enriched by PCR. Each barcoded library product was pooled and subjected to enrichment of targetted genomic sequences, and sequenced on one lane of the Illumina HiSeq 2000 (Illumina, Inc., San Diego, CA, U.S.A.) with paired-end 100 bases read length. Illumina pipeline (version 1.3.4) was utilized to perform bioinformatic analysis of reads mapping, mutations detection (including polymorphisms, SNVs, and small indels) and annotation. All sequencing results were analyzed using nucleotide BLAST (http://blast.ncbi.nlm.nih.gov/Blast.cgi) software to compare with the standard sequences in the database. Mutations of an allele frequency ≥1% in dbSNP, HapMap, 1000G ASN AF, ESP6500 AF, ExAC, Genome1000 and an internal control database from the Beijing Genomics Institute were filtered out. Blood samples were also drawn from 100 unrelated healthy subjects to confirm the mutation was not polymorphisms. We further analyzed the pathogenicity of the mutation using Muation Taster (http://www.mutationtaster.org/) and Uniprot software (http://uniprot.org/).

Mutation in exon 5 of *P4HB* was further confirmed by PCR. The web-based Primer 3 (http://bioinfo.ut.ee/primer3-0.4.0/) was used to design the primers for Sanger sequencing from the genomic sequence (NG_042033.1): forward 5 ′-CTGCTGGCTGCTGTGACTT-3′ and reverse 5′-CAATGGCCTCCAATGTCAG-3′. Following the conditions: initial denaturation at 95°C for 3 min, followed by 35 cycles at 95°C for 30 s, 60.5°C for 30 s, and 72°C for 60 s, PCR was conducted by TaqMix DNA polymerase (Biomed, China) with its standard buffer. PCR products were sequenced by an ABI 377DNA automated sequencer with dye terminator cycle sequencing kits (Applied Biosystems).

### 3D modeling of PDI

Swiss model software (https://www.swissmodel.expasy.org/) was used to build the 3D structure of the PDI. Subsequently, the protein change induced by the mutation was investigated by building the His231Pro mutant of PDI with mutagenesis module of PyMoL software (http://www.pymol.org/).

### Treatment and follow-up

The proband received alendronate (FOSAMAX^®^, Merck Sharp & Dohme Ltd., U.K.) 70 mg/week for 1 year and switched to infusion of zoledronic acid (Aclasta^®^, Novartis Pharma Schweiz AG, Switzerland) 5 mg/year for 2 years and then entered the drug holiday for 1 year. Calcium (500 mg/day) and calcitriol (0.25 ug/2 days) were prescribed during the treatment. To evaluate the effects of bisphosphonates, the change of bone turnover biomarkers and BMD were detected every 6–12 months. All adverse events of the proband were recorded in detail during the treatment.

## Results

### Phenotypes of the proband and her parents

**Figure 2:**
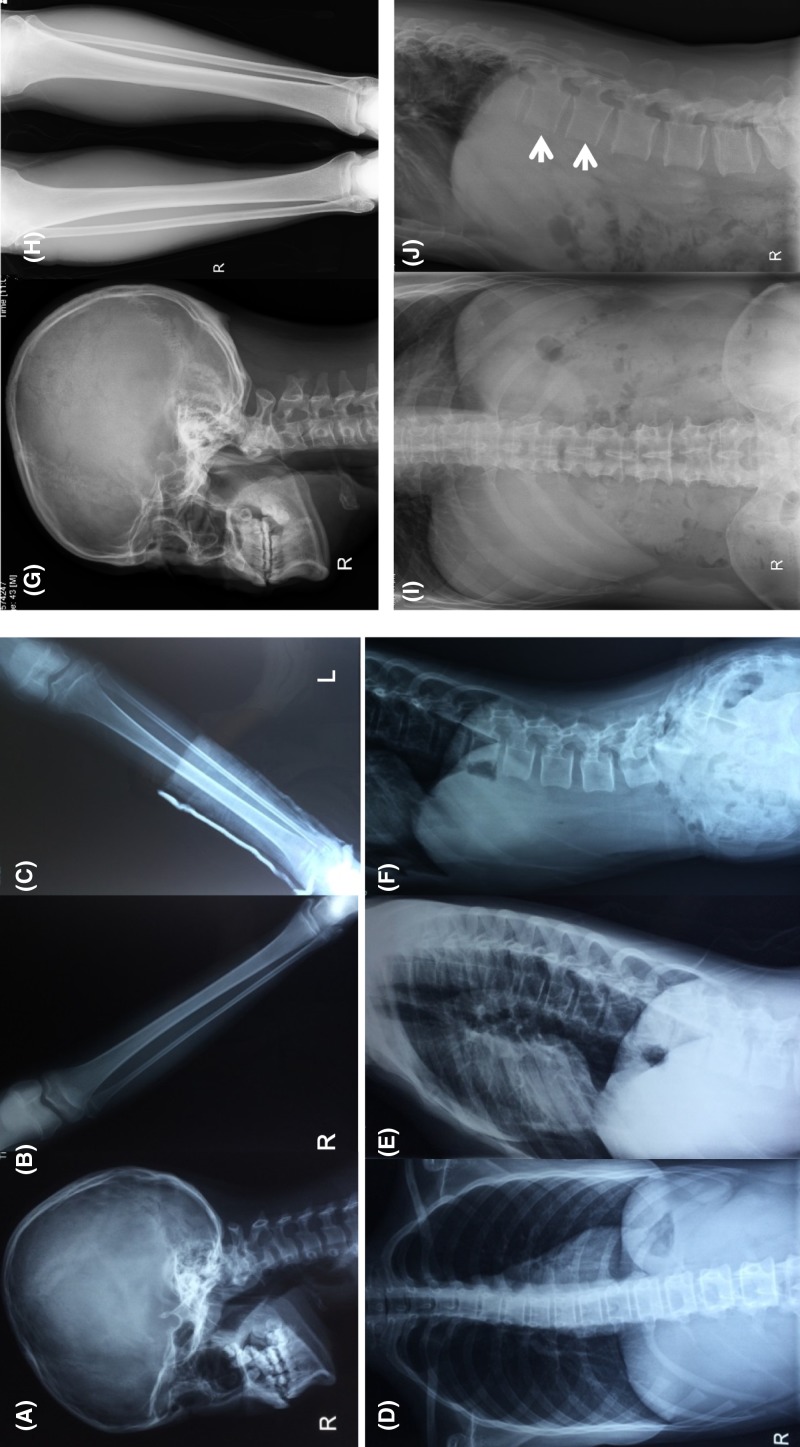
X-ray films of bone of the proband and her father (**A–F**) Imaging features of the proband; (A) no wormian bones at the occipital region; (B,C) slender long bone with thin cortices; (D–F) slight scoliosis of the spine. (**G–J**) Imaging features of the father; (G) no wormian bones at the occipital region; (H) no abnormities of lower extremities; (I–J) slight vertebral wedge changes of the spine: the vertebral morphometry revealed that the reduction of the anterior height of T12 and L1 were 21.5 and 22.2% compared with the posterior height.

The proband was a girl of a non-consanguineous family. She was born full-term with a birth weight of 3300 g. She had no skeletal deformities at birth, with normal cognitive and motor development. Since 6 years old, she was suffered from five non-traumatic fractures of bilateral humerus, left wrist, left ankle, and left calcaneus. At the age of 12, her height was 157 cm with a Z score of 0.72 and her weight was 36 kg with a Z score of −0.54. Physical examination revealed bluish gray sclera and ligamentous laxity, but without bone deformity and dentinogenesis imperfecta. Serum levels of Ca, P, ALP, and PTH were normal. We could not judge if her β-CTX level was normal because the normal range of β-CTX was unavailable in Chinese children. The serum 25OHD level was 10.2 ng/ml, which indicated vitamin D deficiency. BMD value was 0.780 g/cm^2^ (Z score: −0.31) at lumbar spine and 0.646 g/cm^2^ (Z score: −1.6) at femoral neck. X-ray films revealed slender long bone with thin cortices and slight scoliosis of the spine (Figure 2A–F). No wormian bone was observed.

The father was 43 years old with the height of 179 cm. He suffered from one fragility fracture at the fourth metacarpal of the right hand at 30 years old. Physical examination revealed bluish gray sclera without ligamentous laxity, bone deformity, and dentinogenesis imperfecta. BMD Z score was 0.5 at lumbar spine and 0.0 at the femoral neck. Vertebral morphometry revealed slight wedge changes of thoracic and lumbar vertebras (Figure 2G–J). The mother was 41 years old with BMD Z score both 0.2 at lumbar spine and femoral neck. She did not experience fracture. The phenotypes of the family were shown in Table 1.

**Table 1:** Clinical characteristic, biochemical parameters, and BMD of the proband and her parents

	Proband	Father	Mother	Reference range
Age at the first visit (year)	12	43	41	/
Ht(cm)	157	179	161	/
Wt(kg)	35	80	64	/
ALT(U/l)	10	81	NA	5–40
Cr(umol/l)	34	81	NA	18–88
Ca(mmol/l)	2.42	2.33	NA	2.13–2.70
P(mmol/l)	1.45	1.14	NA	1.29–1.94*; 0.81–1.45^#^
ALP(U/l)	162	64	NA	42–390*; 50–135^#^
β-CTX(ng/ml)	1.20	0.135	NA	0.21–0.44^#^
25OHD(ng/ml)	10.2	25.6	NA	30–60
PTH(pg/ml)	34.6	55.6	NA	12–68
LS-BMD(g/cm^2^)	0.780	1.272	1.212	/
LS-BMD Z score	−0.3	0.5	0.2	/
FN-BMD(g/cm^2^)	0.646	0.940	0.978	/
FN-BMD Z score	−1.6	0.0	0.2	/

* The normal range for serum P, ALP in children of 2–18 years old was 1.29–1.94 mmol/l and 42–390 U/l, respectively.

# The normal range for serum P, ALP and β-CTX in adults was 0.81–1.45 mmol/l, 50–135 U/l, and 0.21–0.44 ng/ml, respectively. β-CTX, β-isomerized carboxy-telopeptide of type I collagen, 25OHD, 25 hydroxy-vitamin D; Ca, Serum calcium; FN, femoral neck; Ht, height, LS, lumbar spine; P, Serum phosphate; NA, not available; Wt, weight.

### Mutation in *P4HB*

A novel heterozygous missense mutation in exon 5 of *P4HB* (c.692A>C) was identified in the proband and her father (Figure 1B). This mutation led to histidine replaced by a proline at position 231 in PDI. There was no frequency record of the mutation in dbSNP, HapMap, 1000G ASN AF, ESP6500 AF, ExAC, genome1000, and an internal control database from the Beijing Genomics Institute. It was damaging in prediction of Mutation Taster. Moreover, the affected amino acid residue was highly conserved in PDI amongst different species (Figure 1C), which indicated the function of this residue was important throughout evolution. The change of amino acid of PDI in 3D structure induced by the mutation in *P4HB* was shown in Figure 1D.

The *P4HB* mutation identified in our patients was absent from the 100 healthy controls and did not match polymorphisms in any public database. No mutation was identified in other candidate genes of OI in our patients.

### Effects of BPs on bone of the patient

**Figure 3:**
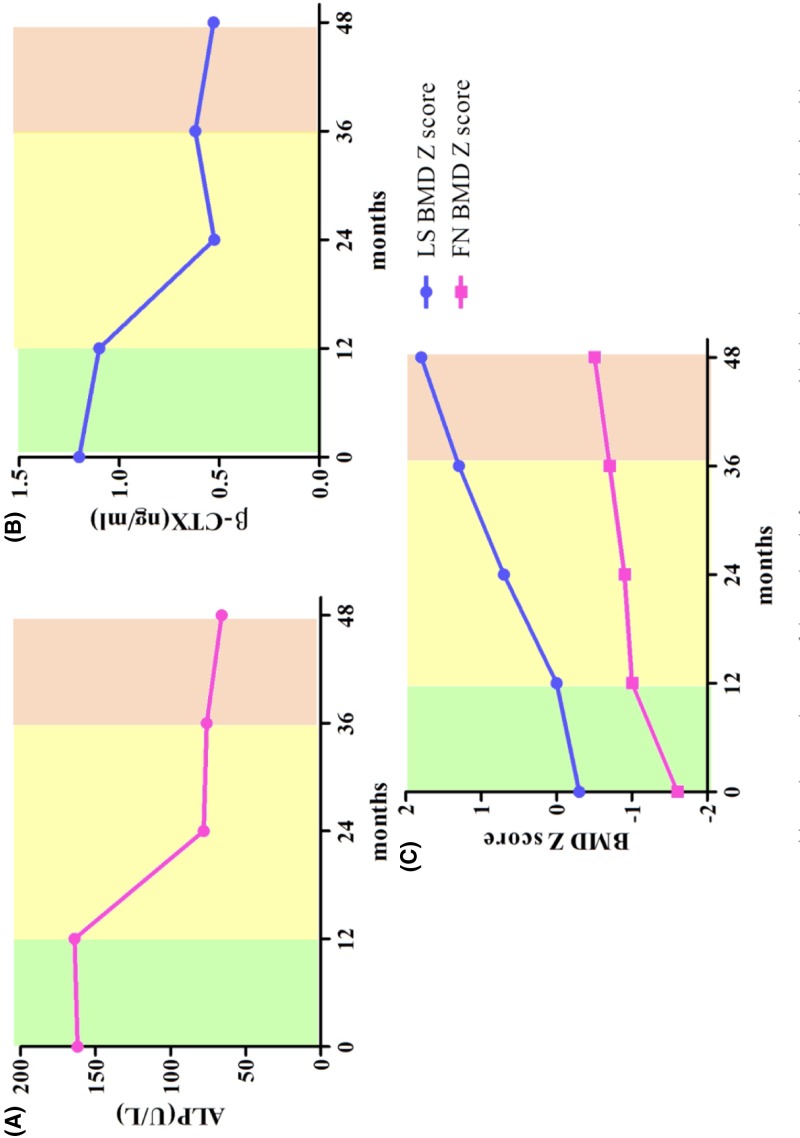
Changes of bone turnover biomarkers and BMD of the proband after treatment with alendronate and zoledronic acid (**A**) Changes in ALP of the proband after bisphosphonates treatment. (**B**) Changes in β-CTX of the proband after bisphosphonates treatment. (**C**) Changes in BMD at lumbar spine and femoral neck of the proband after bisphosphonates treatment.

As shown in Figure 3, after 12 months of treatment with alendronate, serum β-CTX level of the proband decreased by 8.3%. Then serum ALP and β-CTX levels of the proband were significantly decreased by 43.5 and 53.7% after 24 months of zoledronic acid treatment and remained at a low level during the drug holiday. During 3 years treatment of BPs, the BMD of the proband significantly increased by 40.9% at lumbar spine and by 21.2% at femoral neck, with her BMD Z score increasing from −0.3 to 1.8 at lumbar spine and from −1.6 to −0.5 at femoral neck, respectively. Moreover, the height of the proband arrived 168 cm (Z score: 1.46) when she was 16 years old. During the treatment of bisphosphonates, no new fracture occurred. The proband did not present with upper abdominal discomfort and acid reflux, dizziness, and hepatic or renal function abnormity during taking alendronate. She had no fever, myalgia, nausea, vomiting, dizziness, hypocalcemia, hypophosphatemia, and abnormal liver and kidney functions when she switched to treatment of zoledronic acid from alendronate.

## Discussion

We reported for the first time that a novel heterozygous missense mutation in *P4HB* gene (c.692A>C in exon 5) could lead to mild OI. The phenotypes of the proband with this mutation included recurrent fractures, slight scoliosis of spine, slender long bone with thin cortices, and bluish gray sclera, ligamentous laxity. Her father only suffered from one fragility fracture, slight wedge changes in thoracic and lumbar vertebras with bluish gray sclera. Bisphosphonates were effective in reducing bone resorption and increasing BMD of the proband.

The exact mechanism of mutations in *P4HB* causing OI had not been fully illuminated. PDI, encoded by *P4HB*, was a molecular chaperone locating in endoplasmic reticulum (ER) with the capacity to catalyze disulfide bond formation, breakage, and rearrangement in all non-native protein and peptide substrates including type I procollagen [25–27]. PDI played roles as a chaperone with the ability of redox catalyst, which prevented type I procollagen aggregation in the ER [28]. In fibroblasts of patients with *P4HB* mutation, the vesicular pattern of procollagen type I distribution was significantly increased compared with healthy control [10]. In addition, *P4HB* mutation also increased the expression of heat shock protein 47 in fibroblasts, which indicated ER stress [10]. ER stress could lead to osteoblast malfunction and result in defective bone formation and bone fragility, which had been proved in an OI mouse model [29]. Moreover, PDI also offered supports for the prolyl 4-hydroxylase α subunit as the β subunit. Prolyl 4-hydroxylase could aid α chains of type I procollagen forming the triple helix structure by hydroxylating proline residues on the α chains [18]. *P4HB* mutations would disrupt the formation of type I collagen triple helix structure. Therefore, *P4HB* mutations might result in dysfunction of bone formation through multiple aspects.

**Figure 4:**
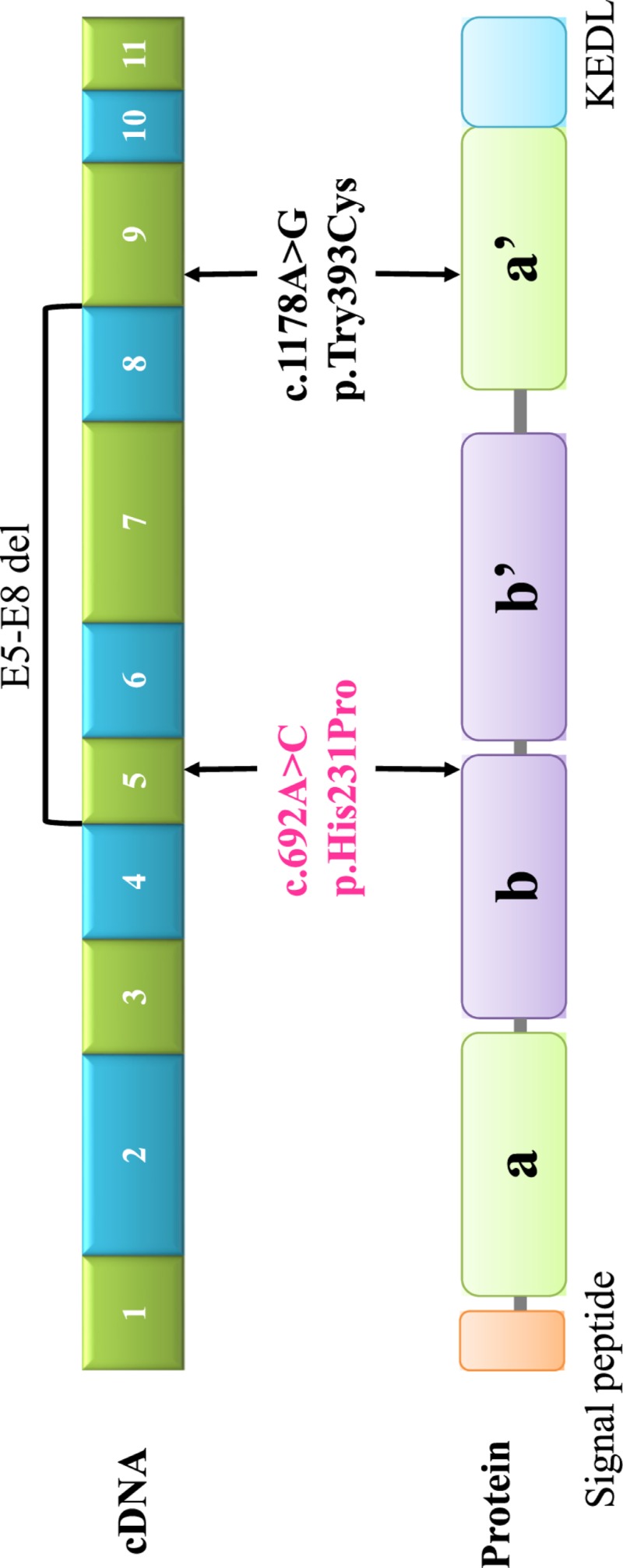
A cartoon of the PDI monomer and distribution of *P4HB* mutations of OI The locations of the exons were aligned relatively to the encoding regions of the PDI The positions of the mutations reported in the previous studies were indicated by black words. The mutation in our patients was indicated by pink words. a and a’ represented disulfide isomerase domain. b and b’ represented substrate binding domain. KEDL (lysine-aspartic acid-glutamic acid-leucine) was an endoplasmic reticulum retention motif.

As shown in Figure 4, including our study, only two heterozygous missense mutations of c.1178A>G in exon 9 and c.692A>C in exon 5 and 1 heterozygous large fragment deletion from exon 5 to 8 in *P4HB* were reported. PDI was currently recognized to have four thioredoxin-like domains (a, b, b’, and a’) with a β-α-β-α-β-α-β-β-α structure and one ER retention motif [16]. The domains a and a’ contained catalytic CXXC motifs with disulfide isomerase activity. Non-catalytic domains b and b’ were involved in substrate recognition and combination [25]. Previous studies revealed that c.1178A>G mutation caused p.Try393Cys of the C-terminal disulfide isomerase domain, which impaired the disulfide isomerase activity of PDI [10–12]. Another study reported a large fragment deletion from exon 5 to 8, which also affected the disulfide isomerase domain [13]. In the present study, we identified a novel heterozygous mutation (c.692A>C) in exon 5 of *P4HB* gene, which caused an amino acid substitution (p.His231Pro). Since the affected amino acid residue was highly conserved in the PDI amongst different species, we speculated that the change of this amino acid would impair the function of PDI. This change was located in the last α helixes of the domain b in PDI, which was responsible for the combination with substrate. It was predicted to cause OI by impairing the combination of PDI with type I procollagen and leading to type I procollagen aggregation in the ER and disorder of its triple helix formation. Due to the exact mechanisms of this mutation lead to OI was not completely clear, the functional experiment was still needed to be completed.

Up to now, including our patients, only seven patients were reported to carry mutations in *P4HB* [10–13]. The phenotypes of our patients were obviously different from previously reported (Table 2). All of the other patients presented severe skeletal phenotypes, including a lower BMD, bowing extremities, popcorn epiphyses or wide epiphyses, craniosynostosis, distinctive facial features, ocular proptosis, and growth retardation, with earlier onset age. But the patients in our study only presented as mild OI, with fewer bone fracture and slight low BMD. The phenotype of patients with *P4HB* mutations exhibited obviously heterogeneous from mild OI to severe CCS. The reasons for the significant phenotypic heterogeneity of OI patients with *P4HB* mutations were unclear. We speculated that different mutation in *P4HB* would affect different domain of PDI, resulting in heterogeneous phenotype of patients. Further studies need to be carried out to elucidate the mechanisms of the heterogeneity in OI patients.

**Table 2:** Genotypes and phenotypes of our patients and previously reported patients with *P4HB* mutations

Phenotype	Patient 1	Patient 2	Patient 3	Patient 4	Patient 5	Patient 6	Patient 7
Gender	Female	Male	Male	Male	Female	Female	Female
Ethnicity	Chinese	Chinese	Caucasian	Caucasian	Caucasian	Thai	Chinese
Age at visit	12 years	43 years	18 years	18 years	3 years	11 months	11 months deletion of exons
Mutation	c.692A>C	c.692A>C	c.1178A>G	c.1178A>G	c.1178A>G	c.1178A>G	5–8
Protein change	p.His231Pro	p.His231Pro	p.Tyr393Cys	p.Tyr393Cys	p.Tyr393Cys	p.Tyr393Cys	NA
Domain	b	b	a’	a’	a’	a’	NA
OI classification	Type I	Type I	CCS	CCS	CCS	CCS	CCS
Age at onset	6 years	30 years	1 month	2 months	6 months	8 months	6 months
Number of peripheral fractures	5	1	NA	NA	NA	NA	NA
Bowing extremities	No	No	Yes	Yes	Yes	Yes	No
Vertebral fractures	No	slight vertebral wedge changes	Yes	Yes	Yes	Yes	No
Scoliosis	Yes	No	Yes	Yes	NA	Yes	NA
Other X-ray features	No	No	Popcorn epiphyses	Popcorn epiphyses	Wide epiphyses	Popcorn epiphyses	Wide epiphyses
LS BMD Z score at baseline	−0.3	0.5	−3.9	−5.1	NA	−6.8	NA
FN BMD Z score at baseline	−1.6	0.0	NA	NA	NA	NA	NA
Bisphosphonate therapy	Alendronate and zoledronic acid	Alendronate	Pamidronate	Pamidronate	Pamidronate	Pamidronate	NA
Wormian bones	No	No	Yes	No	Yes	No	NA
Craniosynostosis	No	No	Yes	Yes	No	Yes	Yes
Communicating hydrocephalus	No	No	Yes	Yes	No	No	NA
Distinctive facial features	No	No	Yes	Yes	Yes	Yes	Yes
Ocular proptosis	No	No	Yes	Yse	Yes	Yes	Yes
Cognitive function	Normal	Normal	Normal	Normal	Normal	Normal	Normal
Sclera	Bluish gray	Bluish gray	White	White	Bluish gray	Bluish gray	White
Dentinogenesis imperfecta	No	No	Yes	No	No	No	NA
Ligamentous laxity	Yes	No	NA	NA	NA	NA	NA
Hearing	Normal	Normal	Normal	Normal	NA	NA	NA
Vision	Normal	Normal	Normal	Normal	NA	NA	NA
Growth retardation	No	No	Yes	Yes	Yes	Yes	Yes
Reference	The present study	The present study	[10]	[10]	[11]	[12]	[13]

FN, femoral neck; LS, lumbar spine; NA, not available.

Bisphosphonates, synthetic analogs of inorganic pyrophosphate, had been turned out to be effective in OI patients [30–32]. Pamidronate was revealed to be effective to increase BMD at lumbar spine, reduce bone fracture incidence, and remodel vertebral bodies in CCS patients caused by *P4HB* mutations [10–12]. As the second-generation and third-generation bisphosphonates, alendronate and zoledronic acid could lead to increases in BMD and reductions in bone resorption biomarkers in OI patients [31]. At present, the dosage, frequency, and course of bisphosphonates treatment in OI patients were still in controversy. Considering the pharmacoeconomic factors and the relatively low bioavailability of alendronate (<1%), we choose a higher dosages of alendronate and zoledronic acid to treat this patient. These dosages had been demonstrated to be safe in our previous study [31]. In our patient, alendronate and zoledronic acid was also effective in decreasing bone turnover biomarkers levels, increasing BMD and with well tolerance. The patient entered the drug holiday after 3 years of bisphosphonates treatment because her BMDs had reached the normal level.

In conclusion, *P4HB* is a novel candidate gene for OI. We identified a novel heterozygous missense mutation of c.692A>C (p.His231Pro) in exon 5 of *P4HB* in a mild OI pedigree for the first time. This mutation might induce OI by impairing the ability of PDI to combine with type I procollagen and leading to disorder of the type I collagen triple helix formation. Bisphosphonates were effective in this extremely rare type OI. Our findings expanded the genotypic and phenotypic spectrum of OI.

## Abbreviations

ALP: alkaline phosphatase
ALT: alanine aminotransferase
BMD: bone mineral density
Ca: calcium
CCS: Cole-Carpenter syndrome
Cr: creatinine
ER: endoplasmic reticulum
FN: femoral neck
LS: lumbar spine
NGS: next generation sequencing
OI: osteogenesis imperfecta
P: phosphate
PCR: polymerase chain reaction
PDI: protein disulfide isomerase
PTH: parathyroid hormone
PUMCH: Peking Union Medical College Hospital
25OHD: 25 hydroxy-vitamin D
β-CTX: β-isomerized carboxy-telopeptide of type I collagen
